# Crystal structure of (*R*)-6-fluoro-2-[(*S*)-oxiran-2-yl]chroman

**DOI:** 10.1107/S205698901501261X

**Published:** 2015-07-08

**Authors:** Yoann Rousselin, Hugo Laureano, Alexandre Clavel

**Affiliations:** a Universite de Bourgogne, ICMUB–UMR6302, 9 avenue Alain Savary, 21000 Dijon, France; b CordenPharma-Synkem, 47 rue de Longvic, 21301 Chenove, France

**Keywords:** crystal structure, nebivolol, absolute configuration

## Abstract

The title compound, C_11_H_11_FO_2_, is a building block in the synthesis of the active pharmaceutical ingredient dl-nebivolol. The synthesis starting from the enanti­omerically pure (*R*)-6-fluoro-4-oxo-3,4-di­hydro-2*H*-chromene-2-carb­oxy­lic acid resulted in a mixture of two stereoisomers, namely (*R*)-6-fluoro-2-[(*S*)-oxiran-2-yl]chroman and (*R*)-6-fluoro-2-[(*R*)-oxiran-2-yl]chroman. The mixture was separated by column chromatography but only one stereoisomer crystallized. The X-ray structure analysis revealed that the solid consisted of the *R,S* isomer. A similar procedure was repeated for (*S*)-6-fluoro-4-oxo-3,4-di­hydro-2*H*-chromene-2-carb­oxy­lic acid and, in this case, the *S,R* isomer was produced as a crystalline solid. Thus, all four stereoisomers of the title epoxide were obtained and their absolute configuration was assigned. The crystal studied was refined as an inversion twin.

## Related literature   

For the synthesis of the enanti­opure title product, see: Jas *et al.* (2011 ▸). For pharmacological properties of nebivolol, see: Van Lommen *et al.* (1990 ▸). For a study of related isomers, see: Horiguchi *et al.* (1997 ▸). For the determination of absolute structure, see: Flack (2003 ▸).
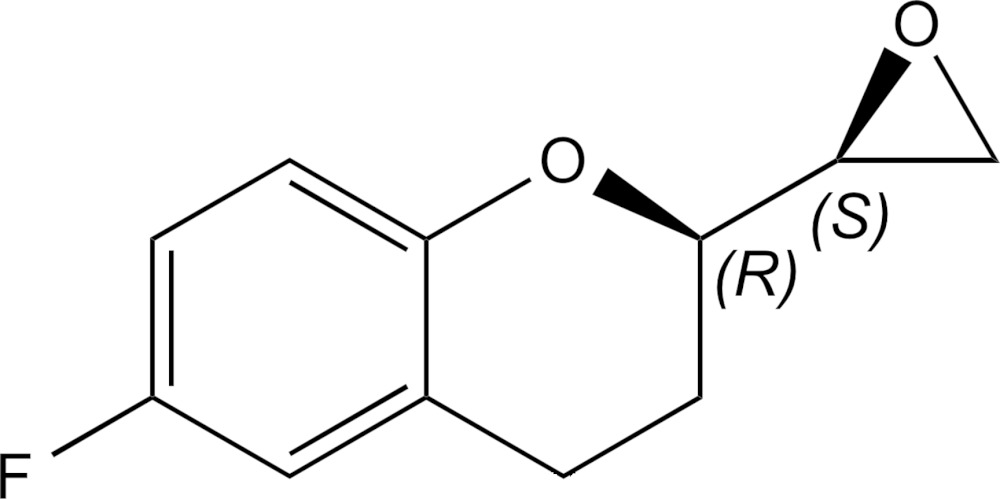


## Experimental   

### Crystal data  

C_11_H_11_FO_2_
*M* *_r_* = 194.20
Monoclinic, 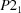

*a* = 9.3742 (3) Å
*b* = 4.76845 (12) Å
*c* = 11.0212 (3) Å
β = 114.202 (4)°
*V* = 449.35 (3) Å^3^
*Z* = 2
Cu *K*α radiation
μ = 0.94 mm^−1^
*T* = 100 K
0.05 × 0.05 × 0.02 mm

### Data collection  

Agilent SuperNova Dual Source diffractometer with an Atlas detector
Absorption correction: multi-scan (*CrysAlis PRO*; Agilent, 2012 ▸) *T*_min_ = 0.64, *T*_max_ = 1
14207 measured reflections
1847 independent reflections
1820 reflections with *I* > 2σ(*I*)
*R*_int_ = 0.050

### Refinement  

*R*[*F* ^2^ > 2σ(*F* ^2^)] = 0.032
*wR*(*F* ^2^) = 0.092
*S* = 1.10
1847 reflections
128 parameters
1 restraint
H-atom parameters constrained
Δρ_max_ = 0.20 e Å^−3^
Δρ_min_ = −0.16 e Å^−3^
Absolute structure: crystal refined as an inversion twin (Flack, 2003 ▸)
Absolute structure parameter: 0.0 (2)

### 

Data collection: *CrysAlis PRO* (Agilent, 2012 ▸); cell refinement: *CrysAlis PRO*; data reduction: *CrysAlis PRO*; program(s) used to solve structure: *SHELXS97* (Sheldrick, 2008 ▸); program(s) used to refine structure: *SHELXL2014* (Sheldrick, 2015 ▸); molecular graphics: *OLEX2* (Dolomanov *et al.*, 2009 ▸); software used to prepare material for publication: *OLEX2*.

## Supplementary Material

Crystal structure: contains datablock(s) I, New_Global_Publ_Block. DOI: 10.1107/S205698901501261X/gk2636sup1.cif

Structure factors: contains datablock(s) I. DOI: 10.1107/S205698901501261X/gk2636Isup2.hkl

Supporting information file. DOI: 10.1107/S205698901501261X/gk2636Isup3.mol

Supporting information file. DOI: 10.1107/S205698901501261X/gk2636Isup4.cml

**Fig. 1.**
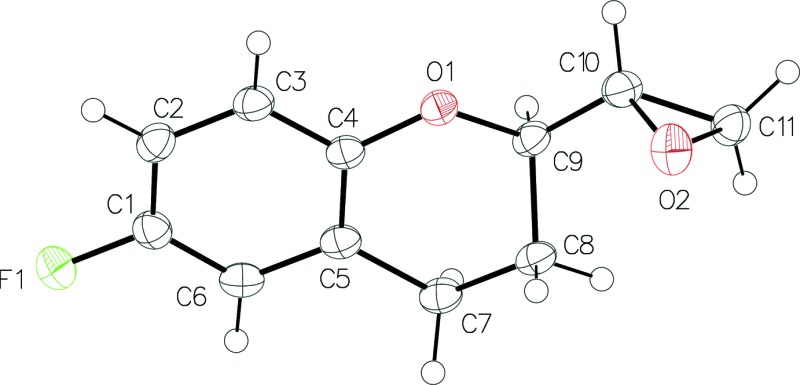
View of the molecular structure of the title compound with 50% probability displacement ellipsoids for the non-hydrogen atoms.

. DOI: 10.1107/S205698901501261X/gk2636fig1.tif
View of the mol­ecular structure of the title compound with 50% probability displacement ellipsoids for the non-hydrogen atoms.

**Fig. 2.**
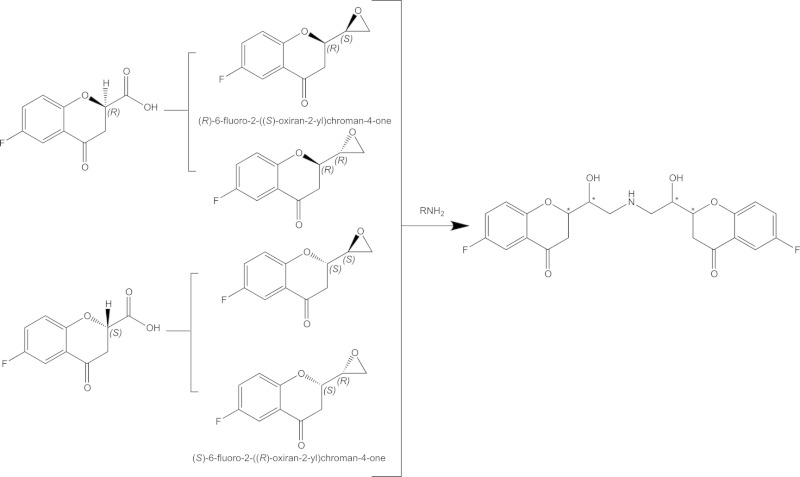
Reaction scheme for the synthesis of nebivolol

. DOI: 10.1107/S205698901501261X/gk2636fig2.tif
Reaction scheme for the synthesis of nebivolol

CCDC reference: 1409735

Additional supporting information:  crystallographic information; 3D view; checkCIF report

## supplementary crystallographic information

### S1. Structural commentary

6-Fluoro-2-(oxiran-2-yl)chroman (Fig. 1) is a building block in the synthesis of *dl*-nebivolol. This active pharmaceutical ingredient is a highly cardioselective vasodilatory β-receptor blocker used in treatment of hypertension.

The synthesis starts from enanti­opure 2-chloro-1-(6-fluoro-chroman-2-yl)-1-ethanol (Jas *et al.*, 2011) that is transformed into enanti­opure 6-fluoro-4-oxo-3,4-di­hydro-2H-chromene-2-carb­oxy­lic acid. Formation of the epoxide from the carb­oxy­lic acid results in a new stereogenic center and a mixture of two stereoisomers is formed from each enanti­opure form: *R,S* and *R,R* from the *R* enanti­omer of the acid and *S,R* and *S,S* from the *S* enanti­omer. The mixtures of stereoisomers can be separated by column chromatography and all four stereoisomers in the reactions can be isolated in a pure form (Fig. 2). These epoxide inter­mediates can be further used in a ring-opening reaction with benzyl­amine to yield, after catalytic hydrogenation, nebivolol isomer with four chiral center.

Of the four stereisomers of the title compound only two form solids, and the remaining two are liquids under normal conditions. X-ray structural analysis from a single crystal of a solid stereoisomer obtained from *(R)*-6-fluoro-4-oxo-3,4-di­hydro-2H-chromene-2-carb­oxy­lic acid revealed that it has R,S configuration at the stereogenic centers (Fig. 1). Crystal data for this stereoisomer are reported in this paper. As expected, X-ray structural analysis of the solid epoxide obtained from the S enanti­omer of the acid revealed the S,R configuration at the stereogenic centers.

### S2. Synthesis and crystallization

(R)-6-Fluoro-2-[(*S*)-oxiran-2-yl]chroman was prepared from enanti­opure 2-chloro-1-(6-fluoro­chroman-2-yl)-1-ethanol according to the procedure reported by Jas *et al.* (2011).

Crystals suitable for X-ray analysis were obtained in a fraction from column chromatography with heptane/ethyl acetate as eluent.

### S3. Refinement

Crystal data, data collection and structure refinement details are summarized in Table 1. All C-bound H atoms were placed at calculated positions and refined as riding on their carriers with C—H = 1.00 Å (methine), 0.99 Å (methyl­ene) or 0.95 Å (aromatic) and with U_iso_(H) = 1.2Ueq(C). TWIN/BASF refinement type in SHELXL-2014 (Sheldrick, 2015) was used to determine absolute configuration from anomalous scattering using the Flack method (Flack, 2003).

### Crystal data

**Table:**

C_11_H_11_FO_2_	*F*(000) = 204
*M**_r_* = 194.20	*D*_x_ = 1.435 Mg m^−^^3^
Monoclinic, *P*2_1_	Cu *K*α radiation, λ = 1.54184 Å
*a* = 9.3742 (3) Å	Cell parameters from 7897 reflections
*b* = 4.76845 (12) Å	θ = 4.4–76.3°
*c* = 11.0212 (3) Å	µ = 0.94 mm^−^^1^
β = 114.202 (4)°	*T* = 100 K
*V* = 449.35 (3) Å^3^	Needle, colourless
*Z* = 2	0.05 × 0.05 × 0.02 mm

### Data collection

**Table:**

Agilent SuperNova Dual Source diffractometer with an Atlas detector	1847 independent reflections
Radiation source: SuperNova (Cu) X-ray Source	1820 reflections with *I* > 2σ(*I*)
Mirror monochromator	*R*_int_ = 0.050
Detector resolution: 5.2474 pixels mm^-1^	θ_max_ = 76.5°, θ_min_ = 4.4°
CCD rotation images, thick slices scans	*h* = −11→11
Absorption correction: multi-scan (*CrysAlis PRO*; Agilent, 2012)	*k* = −5→5
*T*_min_ = 0.64, *T*_max_ = 1	*l* = −13→13
14207 measured reflections	

### Refinement

**Table:**

Refinement on *F*^2^	Primary atom site location: structure-invariant direct methods
Least-squares matrix: full	Hydrogen site location: inferred from neighbouring sites
*R*[*F*^2^ > 2σ(*F*^2^)] = 0.032	H-atom parameters constrained
*wR*(*F*^2^) = 0.092	*w* = 1/[σ^2^(*F*_o_^2^) + (0.0529*P*)^2^ + 0.0952*P*] where *P* = (*F*_o_^2^ + 2*F*_c_^2^)/3
*S* = 1.10	(Δ/σ)_max_ < 0.001
1847 reflections	Δρ_max_ = 0.20 e Å^−^^3^
128 parameters	Δρ_min_ = −0.16 e Å^−^^3^
1 restraint	Absolute structure: crystal refined as an inversion twin (Flack, 2003)
0 constraints	Absolute structure parameter: 0.0 (2)

### Special details

**Table:**

Experimental. Analyzes were recorded in the "Pole Chimie Moleculaire", the technological platform for chemical analysis and molecular synthesis (http://www.wpcm.fr) which relies on the Institute of the Molecular Chemistry of University of Burgundy and Welience"TM", a Burgundy University private subsidiary.^1^H and ^13^C NMR measurements were performed in deuterated methanol on Bruker Avance III, recorded at 500 MHz and 125 MHz, respectively. Chemical shifts (δ) and coupling constants are reported respectively in p.p.m. and hertz (Hz). The optical rotation was measured using a UV Visible Perkin Elmer Lambda 12, polarimeter at 589 nm. High-resolution mass spectrometry (HRMS) was performed in ESI a positive mode. The infrared spectrum (IR) was generated by ATR using a Spectrometer Infrared Avatar 370. A scan range of 4000 - 400 cm^-1^ was used.(2S,2R)-(6-fluoro-2-chromanyl)oxirane:δ(^1^H, DMSO-d6, 300 MHz, ppm): 1.72 (1H, m); 2.00 (1H, m); 2.74 (2H, m); 2.82 (2H, m); 3.17 (1H, ddd); 3.94 (1H, ddd); 6.77 (1H, dd); 6.92 (2H, m).δ(^13^C DMSO-d6, 75.47MHz, ppm): 23.4, 23.7, 44.5, 52.3, 75.3, 113.7 (d, 23.2 Hz), 115.2 (d, 22.3 Hz), 117.2 (d, 8.2 Hz), 123.5(d, 7.5 Hz), 150.1 (d, 1.5 Hz), 156.0(d, 234.8 Hz).[α]^29^_D_ +67.5^o^ (c =1.0 in CHCl_3_)HRMS (ESI) for C_18_H_21_FNO_2_[M+H]^+^ m/z = 195.08158, found m/z = 195.08120.IR (cm^-1^) 3050, 3001, 2951, 1492, 1220Data CCDC-1407326 contains the enantiomer structure of (S)-6-Fluoro-2-[(R)-oxiran-2-yl]chroman. These data can be obtained free of charge from The Cambridge Crystallographic Data Centre via www.ccdc.cam.ac.uk/data_request/cif
Geometry. All e.s.d.'s (except the e.s.d. in the dihedral angle between two l.s. planes) are estimated using the full covariance matrix. The cell e.s.d.'s are taken into account individually in the estimation of e.s.d.'s in distances, angles and torsion angles; correlations between e.s.d.'s in cell parameters are only used when they are defined by crystal symmetry. An approximate (isotropic) treatment of cell e.s.d.'s is used for estimating e.s.d.'s involving l.s. planes.
Refinement. Refined as a 2-component inversion twin.

### Fractional atomic coordinates and isotropic or equivalent isotropic displacement parameters (Å^2^)

**Table:**

	*x*	*y*	*z*	*U*_iso_*/*U*_eq_	
C1	0.7376 (2)	0.5622 (5)	0.50701 (19)	0.0255 (4)	
C6	0.8296 (2)	0.4711 (5)	0.4448 (2)	0.0254 (4)	
H6	0.9095	0.3366	0.4870	0.030*	
C5	0.8061 (2)	0.5754 (4)	0.32005 (19)	0.0226 (4)	
C4	0.6872 (2)	0.7732 (4)	0.26151 (18)	0.0214 (4)	
C3	0.5962 (2)	0.8655 (5)	0.3267 (2)	0.0255 (4)	
H3	0.5165	1.0009	0.2857	0.031*	
C2	0.6216 (2)	0.7604 (5)	0.4512 (2)	0.0261 (4)	
H2	0.5608	0.8231	0.4968	0.031*	
C7	0.9061 (2)	0.4801 (4)	0.24929 (19)	0.0253 (4)	
H7A	0.8759	0.2868	0.2157	0.030*	
H7B	1.0172	0.4770	0.3132	0.030*	
C8	0.8876 (2)	0.6721 (5)	0.13380 (19)	0.0240 (4)	
H8A	0.9306	0.5803	0.0755	0.029*	
H8B	0.9458	0.8488	0.1678	0.029*	
C9	0.7148 (2)	0.7350 (5)	0.05528 (19)	0.0235 (4)	
H9	0.6568	0.5539	0.0267	0.028*	
C10	0.6765 (2)	0.9185 (5)	−0.0645 (2)	0.0271 (4)	
H10	0.5651	0.9814	−0.1082	0.033*	
C11	0.7573 (2)	0.8967 (5)	−0.1530 (2)	0.0286 (5)	
H11A	0.8377	0.7487	−0.1340	0.034*	
H11B	0.6968	0.9384	−0.2485	0.034*	
O1	0.65326 (15)	0.8871 (3)	0.13795 (13)	0.0246 (3)	
O2	0.79031 (19)	1.1254 (4)	−0.06003 (15)	0.0329 (4)	
F1	0.76157 (14)	0.4549 (3)	0.62882 (12)	0.0333 (3)	

### Atomic displacement parameters (Å^2^)

**Table:**

	*U*^11^	*U*^22^	*U*^33^	*U*^12^	*U*^13^	*U*^23^
C1	0.0249 (9)	0.0259 (10)	0.0236 (9)	−0.0037 (8)	0.0079 (7)	−0.0017 (8)
C6	0.0206 (8)	0.0216 (10)	0.0299 (10)	0.0010 (7)	0.0064 (7)	0.0003 (8)
C5	0.0191 (8)	0.0202 (10)	0.0261 (9)	−0.0036 (8)	0.0070 (7)	−0.0049 (8)
C4	0.0185 (8)	0.0201 (10)	0.0238 (9)	−0.0035 (8)	0.0070 (7)	−0.0029 (8)
C3	0.0214 (9)	0.0265 (11)	0.0276 (9)	0.0035 (7)	0.0090 (7)	−0.0011 (8)
C2	0.0245 (9)	0.0274 (11)	0.0282 (10)	−0.0013 (8)	0.0128 (8)	−0.0051 (8)
C7	0.0224 (8)	0.0220 (10)	0.0303 (9)	0.0040 (8)	0.0097 (8)	−0.0013 (8)
C8	0.0180 (8)	0.0252 (10)	0.0288 (9)	0.0010 (7)	0.0096 (7)	−0.0042 (8)
C9	0.0201 (8)	0.0252 (11)	0.0266 (9)	0.0009 (7)	0.0109 (7)	−0.0042 (8)
C10	0.0225 (9)	0.0309 (11)	0.0273 (9)	0.0016 (8)	0.0096 (7)	−0.0015 (9)
C11	0.0272 (9)	0.0315 (11)	0.0286 (9)	−0.0033 (9)	0.0130 (8)	−0.0049 (9)
O1	0.0224 (6)	0.0275 (8)	0.0247 (6)	0.0066 (6)	0.0106 (5)	0.0019 (6)
O2	0.0442 (9)	0.0260 (9)	0.0323 (7)	−0.0047 (7)	0.0195 (6)	−0.0041 (6)
F1	0.0343 (6)	0.0384 (8)	0.0268 (6)	0.0030 (6)	0.0121 (5)	0.0062 (6)

### Geometric parameters (Å, º)

**Table:**

C1—C6	1.374 (3)	C7—C8	1.519 (3)
C1—C2	1.381 (3)	C8—H8A	0.9900
C1—F1	1.366 (2)	C8—H8B	0.9900
C6—H6	0.9500	C8—C9	1.521 (2)
C6—C5	1.392 (3)	C9—H9	1.0000
C5—C4	1.399 (3)	C9—C10	1.500 (3)
C5—C7	1.514 (2)	C9—O1	1.456 (2)
C4—C3	1.393 (3)	C10—H10	1.0000
C4—O1	1.377 (2)	C10—C11	1.463 (3)
C3—H3	0.9500	C10—O2	1.439 (3)
C3—C2	1.387 (3)	C11—H11A	0.9900
C2—H2	0.9500	C11—H11B	0.9900
C7—H7A	0.9900	C11—O2	1.440 (3)
C7—H7B	0.9900		
			
C6—C1—C2	122.35 (18)	C7—C8—H8B	109.9
F1—C1—C6	119.21 (18)	C7—C8—C9	108.92 (16)
F1—C1—C2	118.43 (17)	H8A—C8—H8B	108.3
C1—C6—H6	119.9	C9—C8—H8A	109.9
C1—C6—C5	120.12 (19)	C9—C8—H8B	109.9
C5—C6—H6	119.9	C8—C9—H9	108.9
C6—C5—C4	118.06 (17)	C10—C9—C8	115.48 (16)
C6—C5—C7	121.35 (18)	C10—C9—H9	108.9
C4—C5—C7	120.59 (17)	O1—C9—C8	110.18 (15)
C3—C4—C5	121.04 (18)	O1—C9—H9	108.9
O1—C4—C5	122.66 (16)	O1—C9—C10	104.31 (16)
O1—C4—C3	116.30 (17)	C9—C10—H10	115.1
C4—C3—H3	119.9	C11—C10—C9	122.88 (19)
C2—C3—C4	120.19 (19)	C11—C10—H10	115.1
C2—C3—H3	119.9	O2—C10—C9	117.62 (16)
C1—C2—C3	118.22 (17)	O2—C10—H10	115.1
C1—C2—H2	120.9	O2—C10—C11	59.51 (13)
C3—C2—H2	120.9	C10—C11—H11A	117.8
C5—C7—H7A	109.3	C10—C11—H11B	117.8
C5—C7—H7B	109.3	H11A—C11—H11B	115.0
C5—C7—C8	111.62 (16)	O2—C11—C10	59.42 (13)
H7A—C7—H7B	108.0	O2—C11—H11A	117.8
C8—C7—H7A	109.3	O2—C11—H11B	117.8
C8—C7—H7B	109.3	C4—O1—C9	115.60 (15)
C7—C8—H8A	109.9	C10—O2—C11	61.08 (13)
			
C1—C6—C5—C4	0.0 (3)	C7—C5—C4—O1	1.0 (3)
C1—C6—C5—C7	179.71 (17)	C7—C8—C9—C10	−178.77 (17)
C6—C1—C2—C3	1.3 (3)	C7—C8—C9—O1	63.4 (2)
C6—C5—C4—C3	0.7 (3)	C8—C9—C10—C11	38.9 (3)
C6—C5—C4—O1	−179.29 (18)	C8—C9—C10—O2	−31.0 (3)
C6—C5—C7—C8	−165.51 (18)	C8—C9—O1—C4	−49.5 (2)
C5—C4—C3—C2	−0.4 (3)	C9—C10—C11—O2	−105.1 (2)
C5—C4—O1—C9	17.2 (2)	C9—C10—O2—C11	113.8 (2)
C5—C7—C8—C9	−44.7 (2)	C10—C9—O1—C4	−174.00 (14)
C4—C5—C7—C8	14.2 (2)	O1—C4—C3—C2	179.56 (17)
C4—C3—C2—C1	−0.5 (3)	O1—C9—C10—C11	159.93 (19)
C3—C4—O1—C9	−162.78 (17)	O1—C9—C10—O2	90.0 (2)
C2—C1—C6—C5	−1.0 (3)	F1—C1—C6—C5	179.30 (18)
C7—C5—C4—C3	−178.99 (18)	F1—C1—C2—C3	−179.04 (19)
