# Supplementary figures and images for: Crystal structure of (R)-6-fluoro-2-[(S)-oxiran-2-yl]chroman
Source: Acta Crystallogr E Crystallogr Commun. 2015 Jul 8;71(Pt 8):o552–3. doi: 10.1107/S205698901501261X (PMC4571392; doi:10.1107/S205698901501261X)

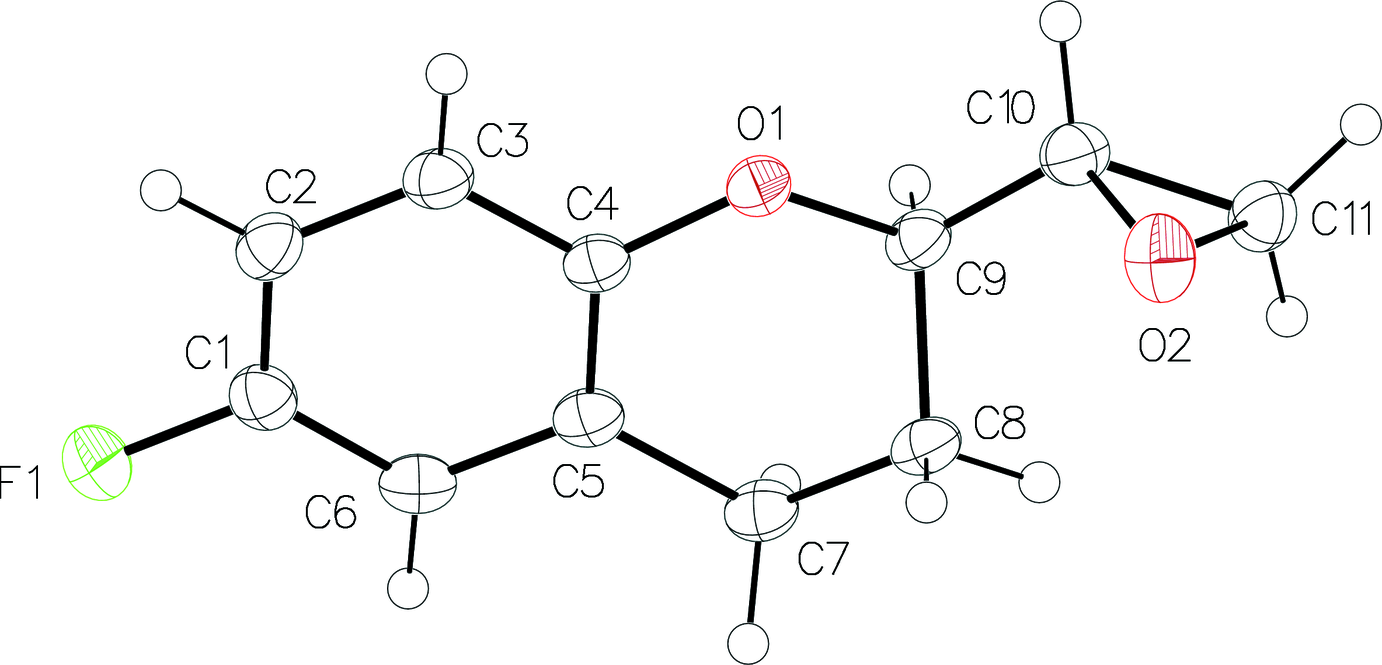

Supplement: Supplementary file 5 [file e-71-0o552-fig1.tif]

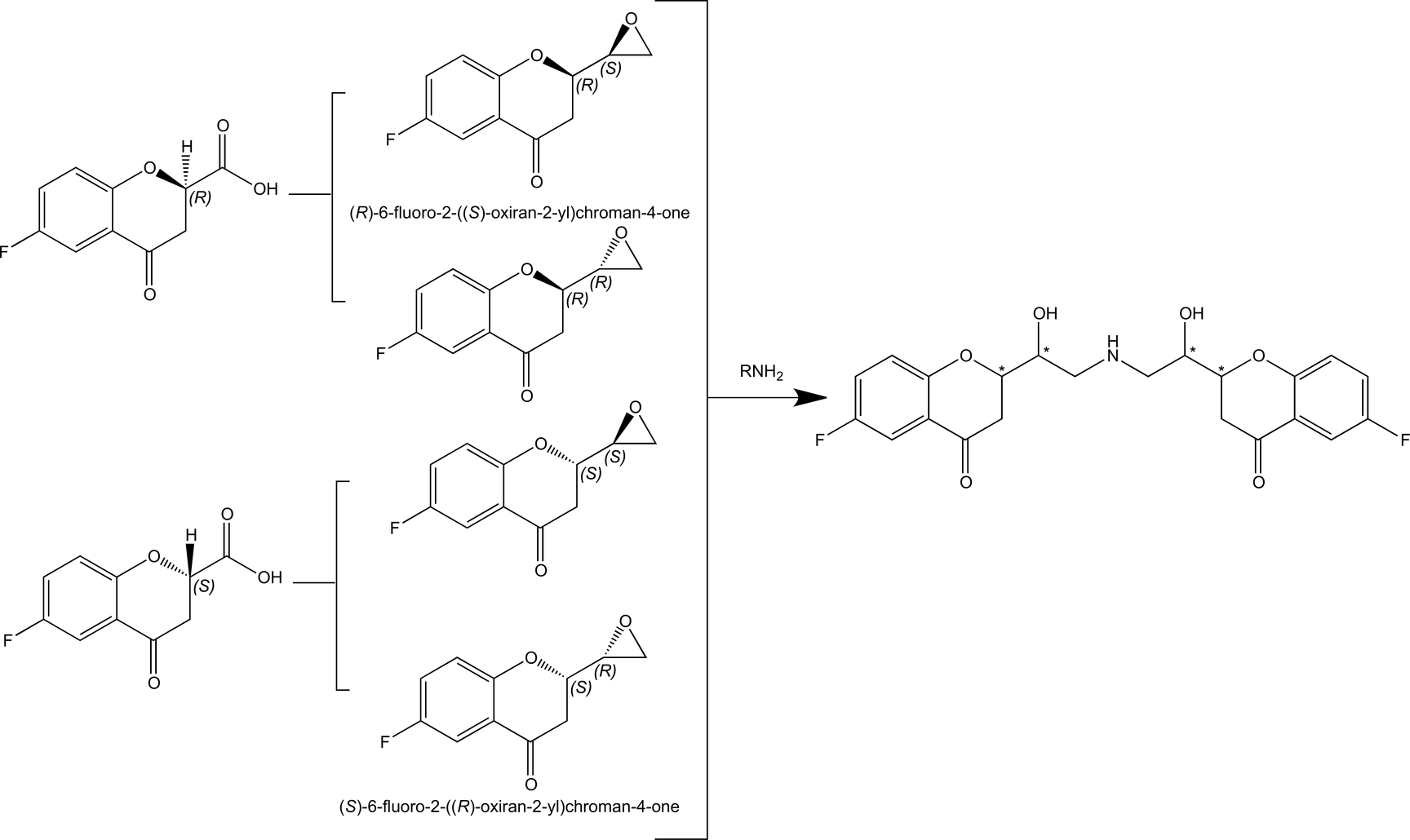

Supplement: Supplementary file 6 [file e-71-0o552-fig2.tif]
